# Determining food industry compliance to mandatory sodium limits: successes and challenges from the South African experience

**DOI:** 10.1017/S1368980023000757

**Published:** 2023-11

**Authors:** Bianca van der Westhuizen, Tamryn Frank, Safura Abdool Karim, Rina Elizabeth C Swart

**Affiliations:** 1 Department of Life and Consumer Sciences, University of South Africa (UNISA), Johannesburg, South Africa; 2 School of Public Health, Faculty of Community and Health Sciences, University of the Western Cape, Cape Town, South Africa; 3 College of Law and Management Studies, University of Kwazulu-Natal, Durban, South Africa; 4 Department of Dietetics and Nutrition, Faculty of Community and Health Sciences, University of the Western Cape, Cape Town, South Africa

**Keywords:** South Africa, Sodium, Regulations, Compliance

## Abstract

**Objective:**

To provide an update on the compliance to the Na reduction regulation (R.214) and to highlight some challenges and successes experienced by South Africa in the implementation of a mandatory Na regulation.

**Design:**

The study design was observational. Nutritional information of packaged food (specified in the R.214 regulation) was collected between February 2019 and September 2020, before and after the implementation date of the final Na targets in the regulation. Six supermarket chains that accounted for more than 50 % of the grocery retailer market share in South Africa were included. The Na content (per 100 g) of products was extracted from photographs. Products were classified according to the thirteen food categories included in R.214. The percentage of targeted food categories that met the pre and post-regulation targets as well as the percentage by which Na limits were exceeded was calculated.

**Setting:**

Low-and-middle-income suburbs in Cape Town, South Africa.

**Participants:**

N/A.

**Results:**

A total number of 3278 products were analysed. After the final implementation date, none of the categories targeted by the R.214 regulation fully complied. However, nine out of the thirteen food categories targeted by R.214 were above the 70 % compliance mark.

**Conclusions:**

The compliance to R.214 in South Africa is good, although not 100 % compliant. This research also highlights the complexities regarding the monitoring and evaluation of a national regulation. Findings from the current study could aid by providing valuable information to countries in the process of implementing a Na reduction strategy.

The impact of non-communicable diseases (NCD) is well documented around the world with more than 15 million people between the ages of 30 and 69 years dying annually from NCD. Eighty-five percentage of these premature deaths occur in low- and middle-income countries^(1)^. CVD accounts for the majority of NCD deaths with 17·9 million people dying from CVD per year, globally. Hypertension is the most significant risk factor for CVD and contributes more than other disease to the global burden of disease^(2)^.

The most recent ‘Science of Salt’ review confirms the positive association between absolute Na intake and high blood pressure^(3)^. The recent Global Burden of Disease study reported that 4·1 million annual deaths have been attributed to excess Na intake^(2)^. Motivated by these associations, the WHO has recognised the need to restrict salt intake to less than 5g/d or Na intake to less than 2000 mg/d.

Despite the WHO recommendation, the 2021 Global Nutrition Report found that no country is on track to achieve the Na reduction target^(4)^. There is a clear need for all stakeholders to amplify efforts to reduce dietary Na intake to counteract the increase in high blood pressure and cardiovascular-related complications.

It is estimated that about half of daily salt intake in South Africa derives from processed foods, with bread being the greatest contributor to non-discretionary salt intake^(5)^. South Africa faces a rising incidence of hypertension, which has a devastating effect on an already burdened health system. In response to this, the South African government developed and implemented a national Na reduction strategy in 2013 in order to reach the Na target of less than 2000 mg/d, recommended by the WHO, before 2025^(6)^. The Na reduction regulation stemmed out of the previous national Obesity Strategy^(7)^, which included reducing hypertension rates in the country. An important step in the national Na reduction strategy was to implement mandatory targets to reduce non-discretionary salt intake by way of a regulation through the Regulations Relating to the Reduction of Sodium in Certain Foodstuffs and Related Matters of 2013 (R.214), which aimed to limit the Na content of certain processed foods (Table 1)^(8)^. The regulation includes thirteen food categories that were identified to contribute the most to the South Africans’ Na intake.

**Table 1:** Regulation R.214: maximum Na levels allowed in certain foodstuffs in South Africa

Foodstuffs category	Max total Na/100 g Phase 1 target (30 June 2016)	Max total Na/100 g Phase 2 target (30 June 2019)
Bread	400 mg Na	380 mg Na
All breakfast cereals and porridges, whether ready-to-eat, instant or cook up, hot or cold	500 mg Na	400 mg Na
All fat spreads and butter spreads	550 mg Na	450 mg Na
Ready-to-eat savoury snacks, excluding salt-and-vinegar flavoured savoury snacks	800 mg Na	700 mg Na
Flavoured potato crisps, excluding salt-and-vinegar flavoured potato crisps	650 mg Na	550 mg Na
Flavoured, ready-to-eat, savoury snacks and potato crisps – salted and salt-and-vinegar only	1000 mg Na	850 mg Na
Processed meats – cured	1300 mg Na* 30 Mar 2017	1150 mg Na* 30 Apr 2020
Processed meats – uncured	850 mg Na	650 mg Na* 30 Apr 2020
Raw-processed meat sausages (all types) and similar products	800 mg Na* 30 Apr 2020	600 mg Na* 30 Apr 2020
Dry savoury powders (not the instant type)	5500 mg Na	3500 mg Na
Dry gravy powders and savoury sauce powders	3500 mg Na	2000 mg Na
Dry savoury powders with dry instant noodles to be mixed with a liquid	1500 mg Na	800 mg Na
Stock cubes, stock powders, stock granules, stock emulsions, stock pastes or stock jellies	18 000 mg Na	15 000 mg Na

R.214: Regulations Relating to the Reduction of Sodium in Certain Foodstuffs and Related Matters of 2013.

* Na target date amended for these categories.

South Africa was one of the first countries to implement mandatory Na reduction targets in a large variety of foodstuffs, including staple foods such as bread and cereals. The implementation of the reduction was two-phased with the first targeted reduction in Na coming into effect in June 2016 (phase 1) and the second target in June 2019 (phase 2). After stakeholder consultation, the South African Department of Health amended some of the target dates of foodstuffs included in the regulation as indicated in Table 1.

One of the limitations with regard to the enforcement of the regulation (R.214) is accountability. While the regulations were introduced by the South African National Department of Health, the implementation and enforcement of the regulations are the responsibility of designated inspectors^(8)^. These designated inspectors are local inspectors who, in addition to other municipal responsibility, inspect retailers and products being sold and are not linked to the South African Department of Health. There is no ongoing evaluation of the regulation by any government agency; however, contraventions and failure to comply may be identified through routine inspections or through reports and complaints submitted to the South African Department of Health.

It is difficult to assess compliance with the regulations at the national level because enforcement occurs at a local level in particular shops or on the basis of individual complaints. Since there is no public record of contraventions reported, it is problematic to ascertain whether contraventions have been prosecuted by the South African Department and led to convictions. To date and to our knowledge, no penalties have been issued for non-compliance with Na regulation (R.214).

As the South African department does not undertake national or wide-scale monitoring and evaluation of the regulations, academic research studies have been the only mechanism to assess the impact of the regulations. The last monitoring of industry compliance was done in 2017 on phase 1 of the R.214 implementation using a direct Na measurement of the foodstuffs in the regulation^(9)^ and using Na information gathered from the nutrition information panel^(10)^.

Continuous monitoring of Na targets included in the R.214 regulation is not only important to evaluate compliance but is also essential in determining the success of the national Na reduction strategy of South Africa. Monitoring provides valuable information that can improve the strategy in South Africa and contribute to global knowledge pertaining to Na reduction efforts. The aim of this research paper is therefore to provide an update on the compliance of industry to the R.214 regulation given the stricter Na limits of phase 2 as well as to highlight some challenges experienced by South Africa in the implementation of a mandatory Na regulation, from a legal perspective.

## Methods

### Sampling procedures

Nutritional information of packaged food was collected between February 2019 and September 2020. To ensure a representative sample of packaged foods available in the South African marketplace, data collection took place in low-income suburbs (Langa and Khayelitsha) and in a middle-income suburb (Durbanville) in Cape Town, South Africa. Six supermarket chains that accounted for more than 50% of the grocery retailer market share in South Africa were included^(11)^. Fieldworkers took photographs of all packaged food products in the store at the time of data collection. Photographs captured all sides of food containers, including the product name, package size, bar code, ingredients and nutritional information. All packaged foods that were available on the day of data collection were selected and photographed, but for the purpose of this study the packaged foods were grouped into the thirteen categories mentioned in the Na regulation (R.214) and were reported as such.

### Fieldwork and data entry

Trained university graduate fieldworkers with a nutrition-related qualification followed a standardised protocol to capture photographs and enter data. The photographs were stored on Sharepoint®, and Research Electronic Data Capture (REDCap)^(12,13)^ was the platform used to enter extracted data from the photographs and perform quality control checks. The Na content of products in the database was verified by identifying outliers and cross-checking against the original photographs of each product and corrected when possible. If a product was photographed twice, the second incident were excluded from analysis. We made use of product barcodes to identify any repeat products. All data were password protected.

Products were classified according to the thirteen food categories included in R.214. As per the criteria in the regulation, Na values were captured in the ‘as sold’ form (rather than the ‘as consumed’ form). Therefore, data were recorded as per 100 g of product as sold (based on the nutrition information panel) and were used for all thirteen categories. Double data entry was performed of all category classifications, and in cases where there were discrepancies these were resolved by discussion with a third researcher.

No direct comparison was done between products pre- and post-regulation. An overall change to the product categories within R.214 was investigated. Data collection comprised of 3278 products, of which 1887 were collected pre- (before June 2016) and 1391 products were collected post the implementation of the final Na regulation (R.214) targets (after June 2019 or in some cases 2020). Products were determined to be either compliant or non-compliant with the regulation based on the Na cut point for each of the thirteen categories as summarised in Table 1.

### Statistical analysis

Data analyses were performed using STATA (version 15, StataCorp). Na levels per 100 g were obtained for each food product targeted by the Na regulation. Within each of the thirteen categories, the minimum, maximum, mean and median Na content per 100 g were reported. The percentage of targeted food categories that met the pre and post-regulation targets as well as the percentage by which Na limits were exceeded was determined.

## Results

A total number of 3278 products were included in the analysis. The breakfast cereal (534), ready-to-eat savoury snacks (784) and cured processed meat (429) categories had the most food products sampled. Overall, compliance of the food categories was good, with the cereal, noodles and stock categories have above 90 % compliance. The gravy and uncured meat category had low compliance with 2·9 % and 23 %, respectively.

**Fig. 1:**
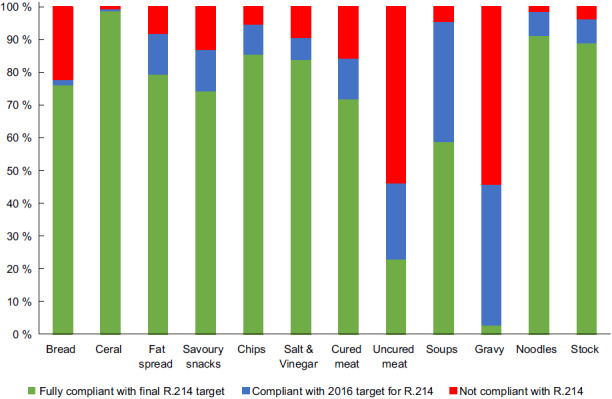
Foods targeted by the R.214 regulation and the percentage compliance to the regulation (post-2019). R.214: Regulations Relating to the Reduction of Sodium in Certain Foodstuffs and Related Matters of 2013

When considering the means reported in Table 2, nine out of the thirteen categories met the final regulation target. Upon closer investigation, Fig. 1 shows the percentage of food products within a food category that complies with the regulation in full, only with the 2016 target or not complying with either of the set targets. Nine out of the thirteen food categories targeted by R.214 were above the 70 % compliance mark. A small proportion of the food categories were not compliant to either of the two target dates, as can be seen in Fig. 1 red bars. The meat sausage category was omitted from the post-regulation analysis because of a small number of food products that were sampled. These food products were all sampled after the initial 2016 target.

**Table 2:** Na content (pre and post-regulation) of foodstuffs categories included in the R.214 regulation

Foodstuffs category	Min	Median	Mean	Max	Na target in R.214	*n* of products
	Na mg/100 g					
Bread						
Post phase 1 regulation target	191	360	375	743	400	105
Post final regulation target	0	357	388	878	380	113
All breakfast cereals and porridges, whether ready-to-eat, instant or cook up, hot or cold						
Post phase 1 regulation target	0	66	132	497	500	219
Post final regulation target	0	138	158	615	400	315
All fat spreads and butter spreads						
Post phase 1 regulation target	0	450	439	826	550	107
Post final regulation target	0	450	400	826	450	97
Ready-to-eat savoury snacks, excluding salt-and-vinegar flavoured savoury snacks						
Post phase 1 regulation target	0	362	636	2680	800	378
Post final regulation target	0	608	556	1820	700	406
Flavoured potato crisps, excluding salt-and-vinegar flavoured potato crisps						
Post phase 1 regulation target	273	562	578	1044	650	84
Post final regulation target	283	517	529	1564	550	76
Flavoured, ready-to-eat, savoury snacks and potato crisps – salted and salt-and-vinegar only						
Post phase 1 regulation target	129	659	702	3382	1000	90
Post final regulation target	0	650	668	2158	850	75
Processed meats – cured						
Post phase 1 regulation target	200	932	1085	3036	1300	397
Post final regulation target	236	988	1071	2213	1150	32
Processed meats – uncured						
Post phase 1 regulation target	75	530	759	2540	850	272
Post final regulation target	68	860	886	2213	650	26
Raw-processed meat sausages (all types) and similar products						
Post phase 1 regulation target	315	681	680	1420	800	34
Post final regulation target	638	838	821	987	600	3
Dry savoury soup powders (not the instant type)						
Post phase 1 regulation target	3568	5364	6136	17 972	5500	80
Post final regulation target	3095	3491	3603	6385	3500	90
Dry gravy powders and savoury sauce powders						
Post phase 1 regulation target	1402	3409	3741	7270	3500	21
Post final regulation target	1402	3591	3603	6385	2000	35
Dry savoury powders with dry instant noodles to be mixed with a liquid						
Post phase 1 regulation target	116	1201	1171	2440	1500	74
Post final regulation target	4	683	671	1890	800	68
Stock cubes, stock powders, stock granules, stock emulsions, stock pastes or stock jellies						
Post phase 1 regulation target	271	17 153	12 667	17 968	18 000	26
Post final regulation target	458	12 355	10 132	30 079	15 000	55

R.214: Regulations Relating to the Reduction of Sodium in Certain Foodstuffs and Related Matters of 2013.

After the final implementation target of 2019 (or 2020 in some cases), not one category had all of the products sampled, fully comply with the regulation, even though in some cases the mean Na per 100 g was compliant to the regulation target.

When studying Table 2, in some cases the mean Na per 100 g increased between the first and final target dates. In most cases even though we reported an increase in the Na content, it was still within the target range indicated by the regulation. In some of the categories like the raw processed meat sausages, the increase reported could be ascribed to the low number of products sample post final regulation target. The uncured processed meat category is another example of a low number of products included post-regulation compared to the post phase 1 regulation target. The food categories with low numbers of food products included should therefore be interpreted with caution.

## Discussion

The majority of the food products within each of the food categories targeted by R.214 were fully compliant with the regulation. Categories with problematic compliance were ‘uncured meat’, ‘raw meat sausage’, ‘dry savoury soup powders’ and ‘dry gravy powders’ categories. Some of the non-compliance could be ascribed to the lower sample size post-regulation and should therefore be interpreted with caution. If these categories continue to show non-compliance to the regulation, consultation with industry partners should be held to address any underlining issues they might experience in these specific categories. Although there was not 100 % compliance, the high level of compliance with the regulation could be ascribed to the mandatory nature of the regulation in South Africa.

Charlton, Langford and Kaldor identified key benefits that mandatory, government-led Na regulation have over voluntary or industry-led initiatives^(14)^. One of the key benefits is that laws apply to all industry actors covered by the scope of the law. This is particularly important in low- and middle-income countries where the food industry is diverse and voluntary commitments from large industry actors may not cover the entire food system^(14)^. In countries where Na limits are voluntary, progress to reduce Na was uneven across different actors^(14)^. This ‘levelling of the playing field’ was identified as a key reason why industry actors were initially supportive of introducing a Na regulation in South Africa^(15)^.

An additional benefit identified by Charlton, Langford and Kaldor is that mandatory Na restrictions are not reliant on changing consumer behaviour to achieve health benefits^(14)^. This benefit of the Na regulation is echoed in South Africa in the findings by Koen *et al*. that non-discretionary Na in processed food is a major source of Na in diets and consumers benefited from the reductions in Na despite 81 % of participants not being aware of the Na regulation^(16)^.

South Africa is at the forefront of countries that implemented mandatory legislation as part of a Na reduction strategy to manage NCD. The results from this study illustrate that while there is not full compliance with the regulation, there is a very high level of compliance across all product categories tested, indicating an overall reduction in the Na within the food supply of South Africa.

South Africa’s high level of compliance stands in contrast to other settings where voluntary limits have been set, such as the UK. The latest review on compliance with the UK’s 2017 Na targets showed a mixed picture. For foods purchased for consumption in-home (retailer own label and manufacturer branded products), just over half of average salt reduction targets have been met which represents no change between 2017 and 2018, although retailers met more targets in 2018 compared with 2017. The shortcomings in meeting these targets appear to be at least partly attributable to the voluntary nature of the UK’s actions. For example, only half of average targets are being met for foods in the fifteen sub-categories contributing the most Na to the diet^(17)^. One must keep in mind that targets set by each country are different and that direct comparisons should be made cautiously.

Other countries that have yet to see success with voluntary Na reduction strategies are Canada and the USA. In 2018, an investigation of the food industry’s progress towards salt reduction targets in Canada revealed that just 14 % of products met their targets. Almost half (48 %) of the products did not achieve any meaningful reduction in salt content and of these, six product categories showed an increase in Na content^(18)^. The USA has similarly seen poor progress. Initially, voluntary Na reduction targets that were set in 2016 by the US Food and Drug Administration^(19)^ were eagerly supported by health charities and non-governmental organisations. The progress on the implementation of these targets was delayed by claims of scientific inaccuracy and opposition from the food industry. Forwarding to March 2019, the National Academies of Sciences, Engineering, and Medicine released a report confirming adult salt intake should be reduced from the current average level of 8·6 g/d to less than 6 g/d^(20)^. No further progress has been reported on Na reduction targets.

In contrast, a recent study reported that the population Na intake of South Africans reduced by 1·16 g salt per day between 2015 and 2018/early 2019, with the median salt intake in their sample group being 6·1 g of salt per day^(21)^. This indicates that, for Na reduction, the mandatory legislation route followed by South Africa is more effective than the voluntary targets followed in the UK, Canada or the USA.

While the regulations have led to significant reductions in Na levels, the findings of this study suggest that better enforcement of the regulations is necessary to ensure that companies are fully compliant with the regulation and that the health benefits of the regulation can be fully realised. There are challenges that arise in implementing mandatory regulations in low- and middle-income countries, and South Africa is no exception. Prior to the implementation, monitoring and evaluation, as well as enforcement were identified by government actors and other stakeholders as key determinants of the effectiveness of the Na regulations^(15)^.

While the Foodstuffs Act grants inspecting, and thus enforcement powers to a wide group of actors including the police force, dedicated foodstuffs inspectors and the revenue service, in practice, enforcement is left to municipal environmental health officers^(8)^. These officers are tasked with enforcement of a large range of regulations and laws, straining already limited capacity. This was identified as a potential challenge in a study conducted by Kaldor prior to the implementation of the Na restrictions where some stakeholders termed the regulations ‘self-regulation by another name’ and de facto self-regulation due to this reliance on environmental health inspectors^(15)^.

In addition, the jurisdiction of these officers operates at a local and district level while a majority of the food producers operate on a national level. As a result, contraventions of the regulations can only be identified at a local level by individual inspectors. This creates two difficulties. The first is that it becomes administratively burdensome for contraventions of the regulations to be individually prosecuted and the localised manner in which contraventions are identified makes consolidating contraventions challenging as well. The second is that individual contraventions offer little disincentive for non-compliant industry actors as the penalties attached to the contraventions are very small, relative to the turnover of these large companies. The maximum penalty that can be levied against a food producer for failing to comply with the regulations is R80 000 (∼USD4900).

The Foodstuffs Act – which the Na regulations fall under – outlines the penalties that attach to contraventions of the regulations^(8)^. These consist of fines (up to R20 000 (∼USD1245), R40 000 (∼USD2490) and R80 000 (∼USD4900) for first, second and third offences respectively) or periods of imprisonment (6, 12 or 24 months for first, second and third offences respectively)^(8)^. These penalties are only levied on an industry actor after they have been convicted of an offence by a court^(8)^.

Given the centrality of monitoring and evaluation as well as enforcement to achieving the potential health benefits of mandatory Na regulations, it is imperative that these challenges be addressed by improving the monitoring and evaluation capacity of inspectors – including the appointment of specific foodstuffs inspectors tasked with ensuring compliance with food-related regulations such as the Na, transfats and other NCD-prevention regulations. To realise meaningful enforcement of the regulations, we recommend that the increased monitoring and evaluation capacity should be coupled with a shift to enforcement of the regulations at a national level. Importantly, individual instances of non-compliance with the regulations should be consolidated and prosecuted as violations associated with all products of a specific food company violating the criteria of the legislation, not solely the few products identified at a particular retailer or in a particular ward. This will remove some of the administrative hurdles to prosecuting violations and also allow for meaningful penalties to attach to non-compliance. In addition, we suggest that penalties attached to non-compliance be restructured to create meaningful disincentives for non-compliance. Other laws in South Africa allow for penalties to be calculated as a percentage of turnover or profit, creating a powerful deterrent against non-compliance. This should be considered for food-related regulations too. Further to that, we recommend that there should be better documentation of transgressions, and more transparency about what steps are taken, and against which manufacturers.

Some of the limitation of this study included that information was reliant on data presented on food packages and not on chemical analysis. This limitation is also highlighted in a South African study by Korff and co-workers when they reported a difference in analysed true Na levels in comparison with what was reported on the label^(22)^. The impact of the global COVID pandemic potentially affected the stocks in stores, hence the smaller product numbers in some food categories post-2019 collection. A clear difference can be seen in the number of food products in pre- and post-regulation within the cured as well as the uncured processed meat categories. This is a result of the small sample size of the post-regulation dataset (due to regulation date being April 2020, and data collection in 2020).

Even though large chain supermarkets (that were all included in data gathering) have control over half of the retail share of the food market^(23)^ (which includes most of the packaged foods sold in South Africa), caution should be exercised in extrapolating results to the wider industry.

Another aspect of this study that could be considered a limitation is that data could not be made publicly available due to agreements with the retailers from which the data were collected. Ongoing negotiations with major retailers and food manufacturers should be pursued to contribute towards a publicly available dataset to promote sharing of data, not only pertaining to the Na regulations but other food-related data.

Future research pertaining to the Na reduction strategy in South Africa should include a comprehensive evaluation on the strategy, as a whole. This would include looking at consumer behaviour change, food supply change and Na intake in the population. This will require stakeholders from government, academia and non-profit organisations all providing input and gathering data to support the Na reduction strategy, in order for other countries to follow and learn from a country like South Africa.

Recognising the limitations, this study is the first to report on compliance with the R.214 post the 2019 target date. Giving insights into the details regarding the monitoring and evaluation of such a regulation is also unique. A recent review indicated that there is an increase in the number of national Na reduction strategies around the world since 2014. More countries are now opting for structural or regulatory implementation strategies including targets for salt levels in foods^(24)^. The current study could aid by providing valuable information to countries in the process of implementing such a strategy. Ultimately, strategies like this will impact the lives of millions globally and should be encouraged by sharing of information.
